# “We Have to Move Quickly to Cement This Willingness for Change”: News Narratives About Declarations of Racism as a Public Health Crisis, 2019–2021

**DOI:** 10.1089/heq.2023.0276

**Published:** 2025-01-08

**Authors:** Hina Mahmood, Pamela Mejia, Katherine Schaff, Catherine Labiran, Xavier Morales, Lori Dorfman

**Affiliations:** ^1^ Berkeley Media Studies Group, Berkeley, California, USA.; ^2^ The Praxis Project, Oakland, California, USA.

**Keywords:** public health, public health policies, racism, news coverage, health inequities, declarations of racism as a public health issue

## Abstract

**Objective:**

To understand how declarations of racism as a public health crisis were portrayed in the news from 2019 to 2021.

**Methods:**

We assessed a national sample of articles (*n* = 127) to see how declarations of racism as a public health crisis were characterized in the news.

**Results:**

Coverage skyrocketed in June 2020 with 800 articles in that month alone, many of which mentioned systemic or structural racism (43% of articles). Government speakers were quoted in 90% of articles while community voices only appeared in 24% of articles.

**Discussion:**

Narratives that center the causes of structural and systemic racism can help inform the public about the health harms of racism and can also report on solutions to achieve health and racial equity that could influence policymakers and the public.

**Health Equity Implications:**

Those proposing new declarations should make concerted efforts to ensure that these declarations generate news coverage, without relying on acts of violence against Black, Indigenous, and People of Color (BIPOC) communities. Public health practitioners, advocates, and officials should center communities most impacted and help them in creating a system that addresses racial and health inequities.

## Introduction 

Our country’s formation, history, and current policy decisions have resulted in unequal access to the social determinants of health (SDOH), which has created profound racial inequities in health.^1^ Structural and systemic racism is “deeply embedded in systems, laws, written or unwritten policies, and entrenched practices and beliefs,” resulting in well-documented racial health inequities in chronic and infectious diseases, maternal and child health, injuries, access to health care, and many other areas of public health.^1^

Decisions at multiple levels of governance at the onset of the global COVID pandemic in early 2020 exacerbated inequities. Black, Latine, and Indigenous populations suffered substantially higher rates of infection, hospitalization, and death compared with White populations,^2^ often because they faced barriers to social factors that support health and were also more likely to work in jobs that do not pay a living wage, to live in crowded conditions, and to perform jobs that did not allow them to work remotely, putting them at higher risk of exposure to COVID-19.^3^

Prior to the onset of the pandemic, some communities and organizations were already working to pass declarations naming racism as a public health crisis. In the summer of 2020, global social movements in response to the police murders of George Floyd Breonna Taylor, Tony McDade, and countless others brought renewed public attention to racial injustice and health inequities. Against this backdrop, as community organizers and public health researchers exposed the disproportionate toll that COVID-19, police violence, and other structural inequities took on Black, Indigenous, and other people of color, several jurisdictions responded by formally declaring racism to be a public health crisis. These declarations are examples of how public health and other government entities are addressing the structural racism that causes health inequities. Passing declarations help educate the public and policymakers about racism as a public health issue and the need to implement policies to rectify these inequities. The American Public Health Association (APHA) analyzed the policies and mechanisms described within declarations that were passed between May 2020 and August 2021.^4^

We wanted to know what was included in public narratives around declarations of racism as a public health crisis to see whether the discourse elevated remedies to long-standing health inequities. If so, public health, government, and community actors can use this visibility to advance work that addresses racial and health inequities.

The news media provide a window into the public discussion around a range of issues, and help shape public understanding as well as the policy agenda.^5,6^ In order to understand media narratives we have to look at how they are framed. The frame is the way an issue is defined, packaged, and presented in the story. When covering stories, journalists select certain arguments, examples, images, messages, and sources to create a picture of the issue.^7^ This selection—or omission—of arguments and voices functions similar to a frame around a photograph, telling us what information is important and what information we can ignore. To understand media frames and narratives about racism as a public health crisis, including how community voices and perspectives appeared, we mapped, categorized, and assessed news coverage from across the country about declarations of racism as a public health crisis.

## Methods

We analyzed the content of print news articles about policies and declarations of racism as a public health crisis published in U.S. newspapers and wire services between July 1, 2019, and December 31, 2021. We assessed (1) when and where declarations of racism as a public health issue appeared in the coverage, (2) how declarations of racism as a public health crisis were framed^7^ in local news, (3) whether community or government voices were included, and (4) if the news coverage described community involvement in the drafting or implementation of the declarations. In our analysis, we assessed how news coverage of the declarations compared with the content of the policies as described by the criteria categorized in the APHA analysis of the declarations.^4^

**FIG. 1.**
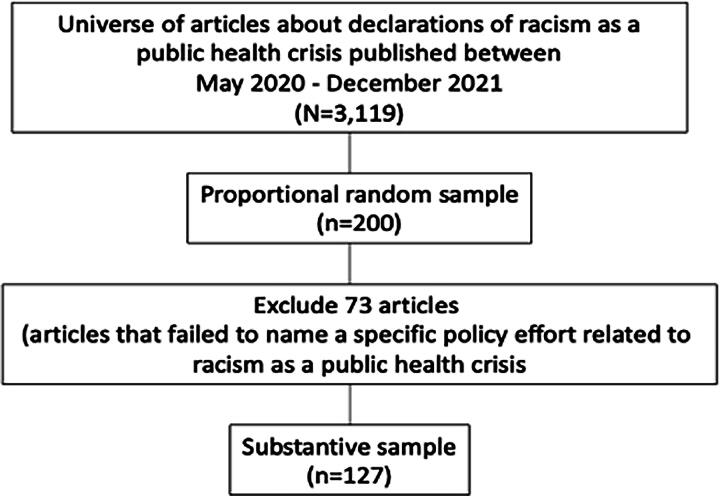
Sampling methods.

We developed a search string to capture all mentions of racism as a public health crisis, including terms such as “racism,” “racial justice,” “public health crisis,” “public health emergency,” and/or “declarations.” We identified a universe of English-language print articles from LexisNexis news archives and collected a proportional random sample by month^8^ to reflect the distribution of news for further analysis and was comprised of articles from states all over the country including California, Florida, Massachusetts, Ohio, as well as national outlets (detailed in Fig. 1). Prior to coding, we removed irrelevant articles (e.g., articles that did not mention declarations of racism as a public health crisis at all).

We developed a coding instrument using an iterative process, drawing on existing literature and previous analyses of equity in the news.^9^ Our coding instrument captured information about where articles were published and when, whether the stories were opinion pieces with a specific perspective or news articles that aimed to present multiple perspectives, and why articles about racism as a public health crisis were published on a given day. We assessed who spoke in the news (i.e., government, community, industry representatives, the medical community, police, and researchers). We also examined how government and community actors’ contributions to the policy process were described. We evaluated how often words related to equity^10^ appeared by searching each article for “equity,” “racial equity,” “equality,” “reconciliation,” “structural racism,” and “systemic racism.”

We trained coders and tested intercoder reliability to ensure that coder alignment did not occur by chance. Specifically, we used an iterative process and coded multiple subsets of articles, then held discussions about any disagreements and adjusted coding decisions until we reached consensus about what the codes included. We achieved satisfactory intercoder reliability scores for all coding variables (Krippendorff’s alpha > 0.8),^11^ and took additional qualitative notes during coding.

In our analysis we also assessed how news coverage of the declarations compared with the real- world content of the policies overall as described by the criteria categorized in the APHA analysis of the declarations. For example, we conducted a qualitative analysis of language in the declarations and compared it with patterns of how they were characterized in the news.

## Results

### When and where did stories about declarations of racism as a public health crisis appear?

**FIG. 2.**
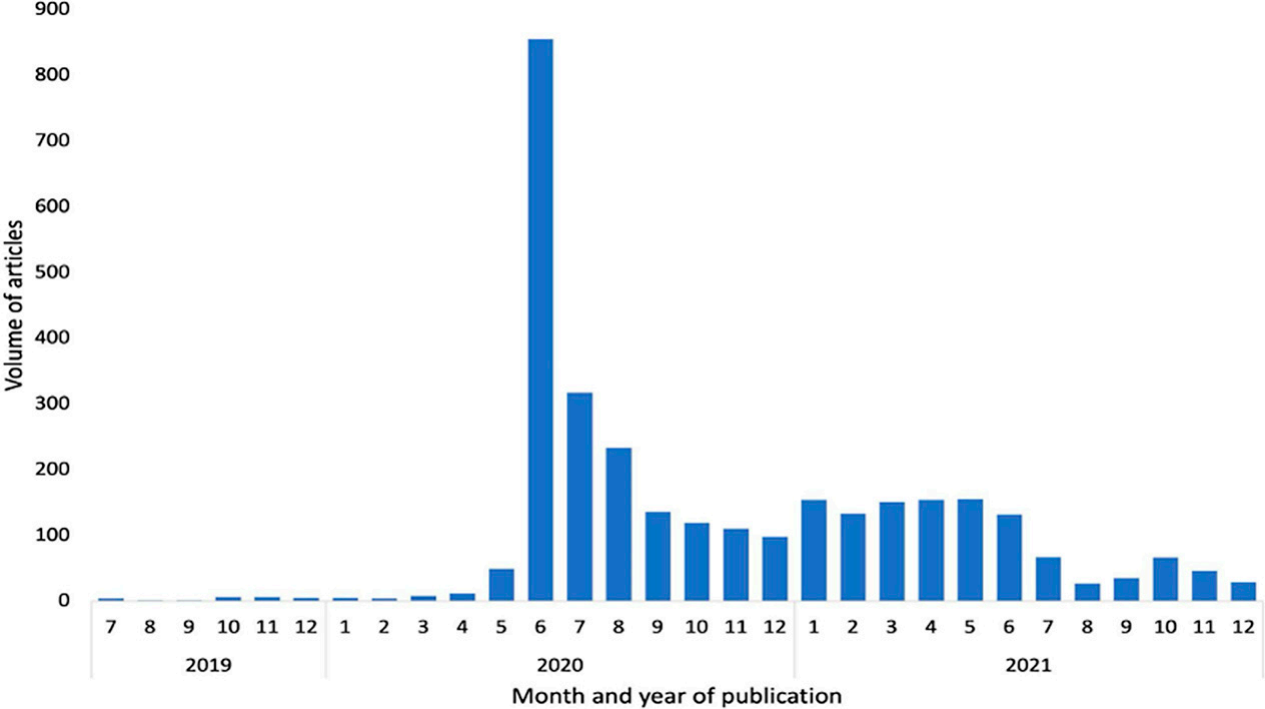
Articles in U.S. news about declarations of racism as a public health issue, July 1, 2019–December 31, 2021 (*n* = 3119 articles). *X*-axis title: Month and year of publication, *Y*-axis title: Volume of articles.

We found 3119 articles about racism as a public health crisis published between May 2020 and December 2021. However, few articles about racism as a public health crisis were published between June 2019 and April 2020 (55 articles). Following the police murder of George Floyd in June 2020 and the subsequent global Black Lives Matter movement protests, news coverage of racism as a public health crisis skyrocketed: 800 articles mentioned declarations in that month alone (detailed in Fig. 2).

We selected a proportional random sample of articles to code in-depth to ensure we captured the range of news narratives across time. After removing irrelevant articles (*n* = 73) we had 127 relevant articles to code.

Almost one-fifth of sampled articles were published from sources based in Ohio, followed by California (13%) and Massachusetts (12%). Almost half of the stories were about declarations at the city level (45%) while one-quarter were about policies at the state level. Almost 60% of stories were about declarations that had passed; the remainder reported on policies that were proposed, under review, or had been rejected.

### How were declarations of racism as a public health crisis framed in local news coverage?

Traditional news articles dominated the coverage (80% of articles) compared with opinion pieces, such as letters to the editor or masthead editorials. More opinion pieces included arguments supportive of declarations of racism as a public health crisis than included critical arguments (13% vs. 5%).

Indeed, news coverage in general rarely included arguments against declarations. Less than a fifth of all articles mentioned explicit opposition to declarations: a rare example came from a Republican selectman from Old Lyme, Connecticut, who was quoted saying that he “does not subscribe” to the idea that racism is a public health crisis.

Most stories were in the news because of an action during the policy process (87%) such as the introduction or passage of a declaration. Many stories focused on health inequities exacerbated by COVID (46%), while other news centered around Black Lives Matter protests in the summer of 2020 and beyond (34%). Racism was regularly described as “systemic racism” or “structural racism” (43%).

#### News coverage about the declarations was incomplete

Only 40% of articles detailed specific actions or accountability measures the declarations contained. One story about Milwaukee County, for example, quoted the County Executive, who noted that the ordinance would “[commit] every single employee of Milwaukee County to embracing probably the most robust legal commitment [to racial equity] in the country,” and concluded that “racial equity will be central to county budgeting decisions, appointments to boards and commissions, and [there will be] continued racial equity training at the county.”^12^

APHA’s analysis of the declarations allowed us to compare news depictions of the declarations with the content of the policies.^4^ APHA’s analysis suggests that the aspiration behind many of the declarations was to engage and center community voices in the policy process. The report documents that the declarations often included language that reflected goals of (1) engaging actively and authentically with communities of color, (2) working in collaboration or partnership with others, (3) addressing systemic racism or improving health in communities of color, and (4) partnering with diverse sectors or stakeholders. Language that reflects these types of community engagement occurred 208 times in the text of the 198 declarations in the APHA analysis^4^ but did not appear regularly in news coverage about the declarations. In news coverage of the declarations community voices were rarely included let alone highlighted in stories, and articles rarely lifted up examples of jurisdictions implementing their declarations. We found that less than half of the articles mentioned concrete actions for implementing declarations, while the APHA report showed that action steps for implementation were part of most declarations (mentioned 149 times across 198 declarations).^4^

### Were government and community voices included in news about declarations of racism as a public health crisis?

Government speakers, including those from local health departments, appeared in nearly all (90%) articles. Most government speakers quoted were at the state or local level (88% of all articles): one quote came from a county judge who named the need to act quickly and follow the direction of those on the racial justice movement’s front lines, saying, “we have to move quickly to cement this willingness for change …. if it’s a top-down solution without community buy-in, it will be doomed to failure. We need to listen to people who basically think we suck.”^13^

Half of government speakers were not elected officials or were not identified as belonging to a political party. State or local speakers identified as Democrats (quoted in 24% of all articles) appeared more often than local government representatives identified as Republicans (9% of all articles).

### Did the coverage describe community involvement in drafting or implementing declarations of racism as a public health issue?

Many articles (40%) described community members and organizations calling for change prior to the passage of a declaration. However, community residents and organizations were directly quoted in only a quarter of articles as when Bridgeport, Connecticut, resident Vanessa Liles, observed, “as a Black woman, I personally experience the effect of systemic racism daily, but for the low-income public housing community, [the effect] is greater because there are multiple systems that weigh-in on their lives.”^14^

Stories rarely discussed community involvement in the process of *drafting or implementing* the declarations (19%). A rare example of a story that centered community input in policy implementation quoted Ricky Burgess of the Pittsburgh City Council, who noted, “It’s important that we guarantee that African-Americans get to choose how resources are given to African-American communities,” and affirmed, “we can ensure there are some uniquely African-American perspectives on how these funds and solutions are implemented.”^15^

## Discussion

To effectively address racism as a public health crisis, our public narratives about the problem must describe structural and systemic causes; news coverage can help when it informs the public about the health harms of racism and its causes. News coverage can also report on solutions to achieve health and racial equity that could influence policymakers and the public.

Declarations of racism as a public health crisis are one tangible way to put structural and systemic racism on the public agenda. Historically, there has been little news coverage of racial health disparities describing solutions rooted in SDOH^16^; the fact that we found frequent mentions (43% of all articles) of “structural” and “systemic” racism can be seen as progress. However, news coverage discussing the health harms of structural racism was almost exclusively driven by policies passed in response to the uprisings about police violence against Black people rather than in response to ongoing health inequities. While the inclusion of terms like structural racism in mainstream news is crucial, because it brings this concept to a wide audience, public health practitioners and advocates need to generate news coverage that draws attention to the many facets of structural racism and keeps the issue in the news over time, not just after incidents of horrific and egregious violence. News coverage should not only mention structural racism but also expand on it by articulating examples that illustrate how it has impacted marginalized populations whenever possible. Future research could explore different characterizations of structural racism and how it resonates with readers.

We also identified many areas where coverage could be more robust. We found that government voices had a significant presence in the news, yet community voices were infrequently quoted. While there was extensive news coverage about the announcement of declarations, there was far less coverage describing how government agencies elevated or executed the policies once enacted and whether they did so in partnership with community residents and organizations. News coverage rarely focused on the content of the declarations or described who should be involved in forming and implementing them. Our analysis also suggests that news narratives about declarations of racism as a public health crisis gives short shrift to the tangible policy and systems change goals outlined in the text of the declarations. This is important because when concrete actions are not named it may be harder for communities to help government actors hold themselves accountable, and for news consumers to visualize what taking action for racial equity looks like in concrete terms.

The lack of news coverage about community engagement with or perspectives on the declarations, especially from communities most harmed by structural racism, reifies a top-down approach to governance, where government officials are called upon as experts and community members, even those with lived experiences of how current policies and structures have perpetuated harm, are left invisible. Furthermore, racial equity experts note that to achieve racial equity, we must transform our systems, which includes having communities that are most harmed at the forefront of the work.^17^ Achieving racial equity in governance will require including community members in news coverage about the work.

Our study is limited in that we used the LexisNexis research archive, which may not have included every outlet that covered the declarations during the time period we studied. Another limitation of our study was that our assessment focused on print media and did not include video footage, which may have provided additional insights into how communities were portrayed. Print media does not provide a full picture of media coverage as other forms such as TV news and social media occupy a significant portion of the media landscape.^18^ However, print news often sets the agenda for local TV news and print articles are shared widely on social media.

## Health Equity Implications

This study is the first to our knowledge to explore news coverage of declarations of racism as a public health crisis. News coverage influences policy agendas and shapes public understanding of issues; news about declarations of racism as a public health crisis present an opportunity to educate broad audiences about the connection between racism and public health outcomes and how racial equity can create healthier communities.^19^ The news narrative can illuminate public health solutions for policymakers, journalists, and the public.

An important part of addressing racism as a public health crisis is acknowledging racial and health inequities rooted in our country’s long history of and present-day structural racism. While news media is only one part of broader movements to shift narratives, systems, and structures, we are encouraged to note that discussions of structural or systemic racism appeared in almost half of the articles. That said, though increasing acknowledgment of structural racism is encouraging, on its own it is not sufficient: this increase came only on the heels of police violence against Black people instead of, for example, in response to longstanding racial and health inequities that predate the summer of 2020. Government and philanthropy can invest in (1) conducting research that assesses how structural racism is described in the news, (2) helping jurisdictions learn from each other’s best practices in implementing policies in communicating about the declarations,^20^ and (3) building capacity for narrative change and media advocacy to increase and improve coverage that ties racism as a public health crisis to specific policy solutions.

As more jurisdictions across the United States draft and implement declarations that name racism as a public health crisis, those proposing them should make concerted efforts to ensure that these declarations generate news coverage, without relying on acts of violence against BIPOC communities. Public health practitioners, advocates, and officials should clearly explain the rationale for their explicit focus on inequities rooted in structural racism and center communities that have been most harmed in both the process of creating and implementing the declarations and in engaging with the news media to talk about their importance.

## Abbreviations Used

APHA: American Public Health Association
BIPOC: Black, Indigenous, and people of color
SDOH: Social determinants of health
